# Phrynoderma and night blindness

**DOI:** 10.4103/0301-4738.60089

**Published:** 2010

**Authors:** Sowmya Raveendra Murthy, Venkatesh C Prabhakaran

**Affiliations:** 1 Department of paediatric ophthalmology and strabismus and Department of oculoplasty, Prabha Eye Clinic and Vittala International Institute of Ophthalmology, Bangalore, India; 2 Department of paediatric ophthalmology and strabismus, Prabha Eye Clinic and Vittala International Institute of Ophthalmology, Bangalore, India

Dear Editor,

Phrynoderma is traditionally considered to be a skin manifestation of vitamin A deficiency as is night blindness in the eye. However, most recent investigators deny the link between phrynoderma and vitamin A and the association between phrynoderma and night blindness is highly unusual. We report a three-year-old patient who presented with night blindness and was found to have skin lesions typical of phrynoderma. Both conditions completely resolved with vitamin A therapy suggesting its causative role.

Ocular manifestations of vitamin A deficiency are referred to as xerophthalmia, earliest manifestation being night blindness. Others include conjuctival xerosis, Bitot's spots, corneal xerosis, keratomalacia, and corneal scarring.[1,2] Skin lesions in the form of follicular keratosis or phrynoderma have been reported.[1,2] Lesions typically are dry, hard, pigmented papules with 2-5 mm central intrafollicular keratotic plug projecting from hair follicles as horny spines resembling toad skin, hence the name phrynoderma. However, the link between phrynoderma and vitamin A deficiency is controversial and some reports suggest that this condition may be secondary to generalized malnutrition or essential fatty acid deficiency.[2–4] Night blindness associated with phrynoderma appears to be an exceedingly rare occurrence.[1–5]

**Figure 1:**
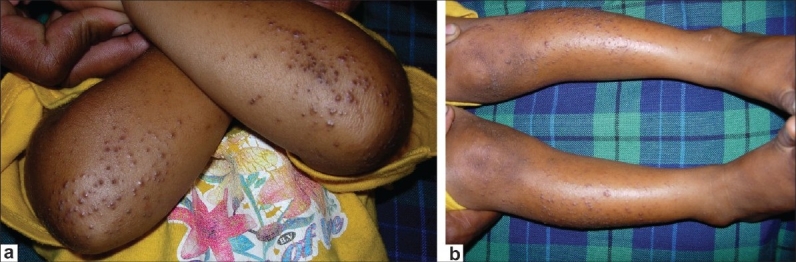
Clinical photographs of the patient's arms (a) and legs (b) illustrating the hard papules on the extensor surfaces typical of phrynoderma

**Figure 2:**
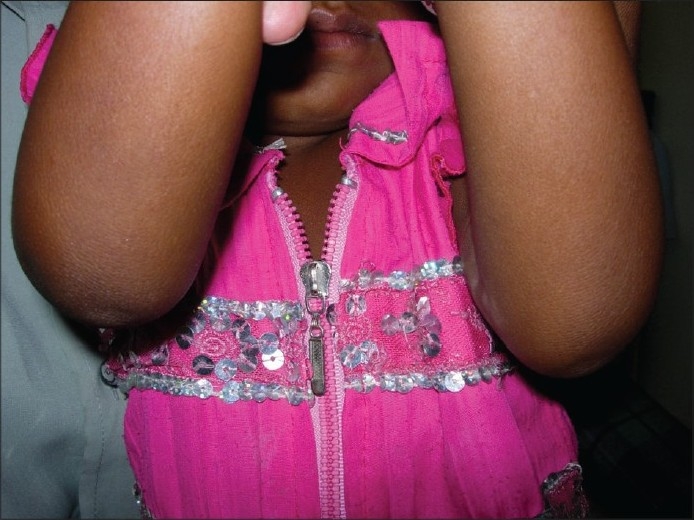
Clinical photograph of the same patient one month following vitamin A therapy

A three-year-old female Indian child presented with history of night blindness of one month duration as ascertained by her parents. On examination, visual acuity appeared to be normal (central, steady, and maintained fixation in both eyes). The conjunctiva appeared lusterless suggesting xerosis. Anterior segment and dilated fundus examination with indirect ophthalmoscope was unremarkable. Further examination revealed dry, horny, papules with central intrafollicular plugging distributed over extensor aspects of elbows, knees, trunk and the back of the child. She also had dry, coarse and lusterless hair. A clinical diagnosis of phrynoderma and vitamin A deficiency was made. Serum vitamin A levels were not estimated as the parents were not willing for the test. Based on clinical findings, vitamin A therapy was considered appropriate. Vitamin A in a dose of 100,000 IU IM was administered on the day of presentation, and the following day. She was referred to a pediatrician for the management of malnutrition. One month follow-up showed complete resolution of skin lesions and parents had also noticed improvement of night blindness confirming the clinical diagnosis of vitamin A deficiency.

The simultaneous occurrence of night blindness and phrynoderma has rarely been reported in literature; to our knowledge, since Nicholls' original report in 1933, only one other case in the literature has documented the association of phrynoderrma and night blindness.[1–5] The earliest ocular manifestation of vitamin A deficiency being night blindness is well known.[1] Phrynoderma is traditionally considered to be a manifestation of vitamin A deficiency,[1] but a number of reports have denied its causative role. Investigators have proposed other causes such as generalized malnutrition and deficiencies of vitamin B, E, and essential fatty acids.[2–4]

Our case demonstrates the simultaneous occurrence of night blindness and phrynoderma, both of which resolved with vitamin A therapy. Though our patient was also malnourished, the effectiveness of vitamin A therapy suggests that at least in our patient, phrynoderma was secondary to vitamin A deficiency.
